# Ssc-miR-130b Enhances Cell Proliferation and Represses Adipogenesis of Primary Cultured Intramuscular Preadipocytes in Pigs

**DOI:** 10.3390/vetsci12040375

**Published:** 2025-04-17

**Authors:** Yunqiu Yang, Yongfang Chen, Lijun Wang, Min Du, Rui Zhang, Yao Lu, Shifeng Pan

**Affiliations:** 1 College of Veterinary Medicine, Yangzhou University, Yangzhou 225009, China; yyq13375299380@163.com (Y.Y.); chenyongfang77@163.com (Y.C.); wlj6379@163.com (L.W.); 2 Department of Animal Sciences, Washington State University, Pullman, WA 99163, USA; min.du@wsu.edu; 3 Meat Processing Key Laboratory of Sichuan Province, Chengdu University, Chengdu 610106, China; zhangrui@cdu.edu.cn; 4 Clinical Medical College, Yangzhou University, Yangzhou 225009, China; lubber0916@163.com; 5 Jiangsu Co-Innovation Center for Prevention and Control of Important Animal Infectious Diseases and Zoonoses, Yangzhou University, Yangzhou 225009, China

**Keywords:** ssc-miR-130b, adipogenic differentiation, proliferation, primary cultured intramuscular preadipocytes, pig

## Abstract

In the efforts towards germplasm innovation of livestock and poultry, strategies to improve meat quality have faced some increasingly challenging and dynamic concerns. Intramuscular fat (IMF) content and backfat thickness are two important traits contributing to meat quality. MicroRNAs (miRNAs)—a class of endogenous noncoding RNAs maintaining cell homeostasis by inhibiting target gene expression—have been proven as critical regulators of body fat deposition, thus affecting farm animal production. Our previous in vitro and in vivo models of pigs have clarified that miR-130b overexpression can obviously suppress adipogenesis of subcutaneous preadipocytes and lower backfat thickness. However, the way miR-130b regulates proliferation and adipogenesis of primary cultured porcine intramuscular preadipocytes (PIMPA) and the underlying mechanism are still unknown. PIMPA derived from longissimus dorsi muscle were employed to examine the role of miR-130b in proliferation and adipogenesis and to further elucidate its underlying mechanism. Lipid deposition in cytoplasm was evaluated by TG quantification and ORO-staining, and EDU-staining was employed to measure cell proliferation. Adipogenic and proliferation-related gene expression were conducted by qPCR and Western blot. MiR-130b overexpression markedly stimulated proliferation of PIMPA by increasing cell cycle-related gene expression. Furthermore, overexpression of miR-130b significantly inhibited adipogenic differentiation of PIMPA, mainly by inhibiting expression of adipogenic differentiation marker genes PPAR-γ and SREBP1. In addition, we proved that miR-130b significantly inhibited expression of PPAR-γ downstream target genes and ultimately repressed adipogenesis. Ssc-miR-130b accelerated proliferation but inhibited adipogenic differentiation of PIMPA, contributing to an enhanced knowledge of the function of ssc-miR-130b in lipid deposition, and providing potential implications for enhancing pork quality.

## Simple Summary

The goal of increasing intramuscular fat (IMF) content and thus improving the meat quality of livestock and poultry involves some increasingly dynamic and challenging concerns. MicroRNAs have proven to be critical regulators of lipid accumulation, among which miR-130b has been demonstrated to inhibit adipogenesis in porcine subcutaneous preadipocytes. In this study, the role and underlying mechanism of miR-130b in regulating cell proliferation and adipogenesis of primary cultured porcine intramuscular preadipocytes (PIMPA) are further studied. Results showed that overexpression of miR-130b accelerated PIMPA proliferation by obviously promoting cell cycle progression, while inhibiting adipogenic differentiation by dramatically reducing PPAR-γ and its downstream target gene expression. Our results indicate that miR-130b plays an inhibitory role in IMF deposition and may serve as a potential candidate for improving meat quality.

## 1. Introduction

In domestic animals, adipose tissue growth is a dominant factor of meat quality and production performance, among which intramuscular fat (IMF) content and backfat thickness are two critical indexes [1]. Especially, the content of IMF significantly influences essential attributes such as tenderness and juiciness [2]. Accordingly, enhancing IMF content remains a key focus in animal science research. IMF deposition is a highly intricate biological process governed by multiple factors [3]. At a cellular level, IMF content is primarily determined by proliferation and adipogenesis of preadipocytes, a population of cells widely distributed within IMF. The preadipocyte number reflects their proliferative capacity, while their differentiation into lipid-laden cells influences lipid accumulation. Together, these processes contribute to the growth and development of adipose tissue [4]. At the molecular level, preadipocyte activity is a tightly regulated and highly intricate process. It is primarily governed by the coordinated interaction of genes related to lipid metabolism, key transcription factors, and epigenetic regulators, all of which play an essential role in orchestrating adipogenesis and fat deposition [5]. Under such circumstances, to clarify the potential mechanism underlying proliferation and adipogenic differentiation of the preadipocyte is of great economic and academic value for enhancing meat quality in both livestock and poultry production.

Accumulating evidence suggest that proliferation and differentiation of preadipocytes involves a highly orchestrated series of intricate biological processes, among which microRNAs (miRNAs) are one group of the most important regulatory factors [6]. As endogenous non-coding small RNAs, MiRNAs inhibit target gene expression [7]. A growing number of studies have previously suggested that miRNAs play an important role in regulating proliferation, differentiation, development, and apoptosis [8]. Moreover, it is proven that miRNAs direct adipocyte fate decisions that are important for proliferation and adipogenic differentiation, which greatly contributes to adipose tissue growth [9]. Recent computational and experimental studies have also shown that miRNAs serve as key regulators in various biological processes related to lipid deposition. Furthermore, more and more studies have highlighted that as crucial epigenetic regulators, miRNAs play a vital role in IMF deposition [10]. In Erhualian pigs, it has been shown that miR-32-5p is a key contributor to IMF accumulation by inhibiting KLF3 [11], whereas miR-381-3p has been shown to suppress IMF deposition via repressing FABP3 [12]. However, the role of miR-130b in controlling proliferation and adipogenesis of porcine intramuscular preadipocytes (PIMPA) and the underlying mechanism remain largely unclear.

It was demonstrated previously that miR-130b expression in plasma of obese mouse models and patients exhibited a strong positive correlation with body mass index (BMI), and the miR-130b secretion in adipocytes significantly increased during fat formation, showing that blood miR-130b was a more reliable indicator predicting metabolic syndrome compared to TG level [13]. Moreover, miR-130b has also been considered as an obesity-related miRNA [14]. Also, it was proven that miR-130 served as an inhibitor of adipogenesis of human preadipocytes. Overexpression of miR-130 significantly reduced lipid accumulation, while inhibition of miR-130 obviously increased lipid accumulation by directly targeting PPAR-γ [15,16]. Furthermore, miR-130b duplex was able to directly suppress KLF3 expression, thereby inhibiting the adipogenic differentiation, fatty acid synthesis, and lipid deposition of goat intramuscular adipocytes [17]. In addition, miR-130a/b overexpression significantly inhibited lipid deposition in bovine intramuscular preadipocyte by targeting PPAR-γ and CYP2U1 [18]. In our previous studies, both in vivo and in vitro experiments in pigs revealed that miR-130b downregulates PPAR-γ expression and suppresses backfat thickness [19]. Our subsequent study revealed that lipid accumulation was significantly decreased by MV-shuttled miR-130b via suppressing PPAR-γ expression in primary cultured porcine adipocytes [20], and this function was further confirmed in HFD-induced obese mice [21]. The aforementioned findings collectively suggest that miR-130b is a potential inhibitor of subcutaneous fat deposition. However, backfat thickness and IMF are two important indexes of pig meat quality; although the inhibition of miR-130b in pig subcutaneous lipid deposition has been largely clarified, the actual role of miR-130b in proliferation and adipogenic differentiation of PIMPA is still largely unknown, and the underlying molecular mechanisms need to be further clarified.

The study utilized PIMPA to further investigate the impact of miR-130b on IMF deposition by regulating proliferation and adipogenic differentiation. MiR-130b mimic and miR-130b inhibitor were used to examine its biological function in the process of adipogenesis and provided evidence that overexpression of miR-130b reduced lipid accumulation during PIMPA differentiation. Suppression of endogenous miR-130b using an inhibitor enhanced adipogenic differentiation. The above results demonstrated firstly that ssc-miR-130b promoted proliferation and inhibited adipogenic differentiation of PIMPA, which provides new understanding to further unravel the role of miR-130b. In summary, our findings have provided a meaningful point of view on miR-130b in porcine IMF deposition, which has potential significance for improving meat quality.

## 2. Material and Methods

### 2.1. Ethics Statements

Experiments involving animals were conducted in compliance with the guidelines for the care and use of laboratory animals established by the Ministry of Science and Technology of the People’s Republic of China (approval no. SYXK-SU-2007-0005) and were approved by Yangzhou University.

### 2.2. Isolation and Culture of PIMPA

In the study, the postnatal Erhualian piglets at 3–7 d of age were anesthetized with injection intraperitoneally of 50 mg/kg body weight pentobarbital sodium followed by exsanguination. The longissimus dorsi muscle samples were meticulously prepared by removing visible blood vessels and connective tissues before being finely chopped into 0.5–1.0 mm^3^ pieces. The primary PIMPA were derived from longissimus dorsi muscle of the piglets by digestion of collagenase, and purification by percoll density gradient centrifugation method. The purified PIMPA were then plated with a 3.0 × 10^4^ cells/well density in 24-well or 6-well plates and maintained at 37 °C in a humidified atmosphere with 5% CO_2_. Before adipogenic differentiation, cells were cultured in growth medium consisting of DMEM/F-12 (Hyclone, Logan, UT, USA) supplemented with 10% fetal bovine serum (FBS) and 1% antibiotic–antimycotic mixture. Culture medium was then changed every 2 days.

### 2.3. Experimental Groups and Adipogenic Differentiation

When 85–90% confluence was reached, the primary cultured PIMPA were randomly divided into four groups: (1) mNC group with miR-130b mimic control transfection; (2) mimic group with miR-130b mimic transfection; (3) iNC group with miR-130b inhibitor control transfection; (4) and inhibitor group with miR-130b inhibitor transfection. To induce adipogenic differentiation, PIMPA were first cultured in differentiation medium (DM) following confluence for two days. The DM was composed of 10% FBS, 1 μM dexamethasone (DEX), 1.7 μM insulin, and 0.5 mM IBMX. Then, the DM was replaced subsequently with DMEM containing 10% FBS as well as 1.7 μM insulin for another two days. Following this, the regular medium was changed every other day until the end of 7 days.

### 2.4. MiR-130b Mimic and Inhibitor Transfection

Primary cultured PIMPA were subjected to serum starvation for 4 h before miR-130b mimic and inhibitor (Suzhou GenePharma Co., Ltd., Suzhou, China) transfection. miR-130b mimic, miR-130b inhibitor, or the corresponding negative controls (NCs) were transfected into PIMPA using ribo Fect™ CP Transfection Kit (Ribobio, Guangzhou, China) at a concentration of 100 nM/well, as suggested by the manufacturer’s instructions. The cells were harvested at 48 h (early differentiation stage) and 7 d (late differentiation stage) after transfection and adipogenic differentiation for further analysis.

### 2.5. Oil Red O (ORO) Staining and Quantification

For ORO staining, mature adipocytes at day 7 of induced differentiation were washed with PBS and fixed in 4% paraformaldehyde for 30 min. Following fixation, the adipocytes were rinsed three times with PBS and stained with a 1% filtered ORO solution (Solarbio, Beijing, China) for 30 min. Excess stain was removed after washing the cells with PBS three times, after which they were examined under an IX53 inverted fluorescence microscope (Olympus, Tokyo, Japan) to identify ORO positively stained adipocytes, which appeared red. For quantification, three images were randomly captured per observation field to determine the ORO-stained area. Additionally, ORO retained in the cells was extracted using 100% isopropanol (*v*/*v*) and quantified by detecting OD510 values.

### 2.6. Total RNA Extraction and qRT-PCR

Total RNA was extracted and reverse transcribed into cDNA. The Applied Biosystems 7500 Real-Time PCR System (Thermo Fisher Scientific, Inc., Shanghai, China.) was used to perform qPCR experiment. The PCR amplification protocol: initial denaturation at 94 °C for 5 s, followed by 40 cycles of annealing at 62 °C for 40 s and extension. All experiments were conducted independently in triplicate. mRNA expression levels were normalized to peptidylprolyl isomerase A (PPIA). Primers used for gene detection were synthesized by Genewiz, Inc. (Suzhou, China), with primer sequences shown in Table 1.

### 2.7. Western Blot

For total protein extraction, protein concentration determination, and electrophoresis, refer to our published studies [19,20,21]. Membranes were then incubated overnight at 4 °C with primary antibodies against PPAR-γ and SREBP-1 diluted in TBST. Following incubation, membranes were incubated with goat anti-rabbit IgG peroxidase-conjugated secondary antibodies (Bioworld Technology, St. Louis Park, MN, USA) at a 1:10,000 dilution. Protein bands were detected by an enhanced chemiluminescence (ECL) system with the LumiGlo substrate (SuperSignal West Pico Trial Kit, Pierce, Rockford, IL, USA). The bands were visualized and captured using the VersaDoc 4000MP imaging system (Bio-Rad, Hercules, CA, USA), and band intensity was automatically quantified using Quantity One software (Bio-Rad), Version 4.3.0. β-actin was served as the internal reference protein, and protein expression levels were shown as relative fold changes compared to mNC or iNC cells, with Table 2 presenting the details of the antibodies used in the study.

### 2.8. Real-Time PCR Quantification of miR-130b

Specific primers used for miR-130b expression quantification were shown in Table 3. RT-qPCR was conducted using an Mx3000P real-time PCR system (Stratagene, Santa Clara, CA, USA). Firstly, genomic DNA contamination of total RNA was removed by treating with RNase-free DNase Kit (Promega, Madison, WI, USA). Subsequently, the purified total RNA (4 μg) was treated with the Poly(A) Tailing Kit (AM1350, Ambion, TX, USA) polyadenylation following the manufacturer instructions.

The total volume of 20 μL polyadenylation reaction consisted of 4 μg RNA (1 μg/μL), 2 μL of 25 mM MnCl_2_, 4 μL of 5× E-PAP buffer, 0.8 μL of E-PAP enzyme, 2 μL of 10 mM ATP, 0.2 pmol each of external controls (E5, E2, and E1), and RNase-free water. After extraction of phenol-chloroform and precipitation of ethanol, the poly(A)-tailed RNA was diluted in diethylpyrocarbonate (DEPC)-treated water.

For cDNA synthesis, 1 μg/μL oligo(dT)-adapter primers were used. The RT1 reaction contained a 10 μL volume of poly(A)-tailed RNA (2 μg), 1 μL oligo(dT)-adapter primer, and DEPC-treated water, and was incubated for 5 min at 70 °C. The subsequent RT2 reaction (25 μL total volume) included RT1 reaction product (10 μL), 10 mM dNTPs (1.25 μL), M-MLV 5× buffer (5 μL), M-MLV reverse transcriptase (1 μL, 200 U/μL), RNase inhibitor (0.5 μL, 40 U/μL), and DEPC water. The cDNA synthesis reaction was conducted by incubation at 42 °C for 1 h. The PCR reaction (25 μL in total volume) consisted of 2 μL RT2 product, 2 μL primers (10 μM), 12.5 μL SYBR Green PCR Master Mix (TaKaRa, Tokyo, Japan), and 8.5 μL sterile deionized water. E5 small nuclear RNA expression was used for normalize miR-130b expression. Data are presented as fold changes relative to mNC or iNC cells, using the 2^−ΔΔCt^ method.

### 2.9. The Effect of miR-130b on PIMPA Proliferation

When PIMPA reached 85–90% confluence, cells were transfected with miR-130b inhibitor, miR-130b mimic, or their respective negative controls (iNC and mNC) using Lipo2000 transfection reagent (Invitrogen Life Technologies, Carlsbad, CA, USA). Two days post-transfection, the CCK-8 Cell Counting Kit (Vazyme, Nanjing, China) was used for PIMPA proliferation, and Varioskan LUX Multimode Reader (Thermo Fisher Scientific, Waltham, MA, USA) was used for detection OD450 values.

To evaluate the proliferation-related gene expression, p27, cyclin B1 (CCNB1), and proliferating cell nuclear antigen (PCNA) mRNA levels were quantified by RT-qPCR in triplicate.

Additionally, DNA synthesis and cell proliferation were analyzed by the EdU incorporation assay. After 2 days of transfection, PIMPA were incubated with 10 μM EdU reagent A (Beyotime, Beijing, China) in cell medium at 37 °C for 2 h. After being fixed with 4% paraformaldehyde, PIMPA were then incubated for 30 min with Click Reaction Solution (Beyotime, Beijing, China). Hoechst 33342 was used to counterstain nuclei for 30 min, and fluorescence signals were observed by an IX53 microscope, and at least three visual fields were randomly taken from each sample to determine the total number of PIMPA and EdU-positive PIMPA for statistical analysis.

### 2.10. The Effect of miR-130b on the Differentiation of PIMPA

To investigate the role of miR-130b in regulating PIMPA adipogenesis, RNA was extracted from adipocytes induced to differentiate into adipocytes for 7 days. The expression of miR-130b, and PPAR-γ, a known miR-130b target gene, as well as key markers associated with differentiation and lipid metabolism—including fatty acid synthase (FAS), glucocorticoid receptor (GR), fat mass and obesity-associated protein (FTO), Perilipin, 11β-hydroxysteroid dehydrogenase type 1 (11β-HSD1), stearoyl-CoA desaturase 1 (SCD-1), and PPAR-α—were evaluated by RT-qPCR.

### 2.11. Statistical Analysis

Statistical analyses were performed by SPSS 26.0 software, and all data are shown as mean ± SEM. Differences between two groups were assessed using Student’s *t*-test (unpaired, parametric). For multiple group comparisons, one-way ANOVA was conducted, followed by Duncan’s test and Bonferroni correction. A *p*-value lower than 0.05 represents statistically significant differences.

## 3. Results

### 3.1. Isolation and Identification of PIMPA

**Figure 1:**
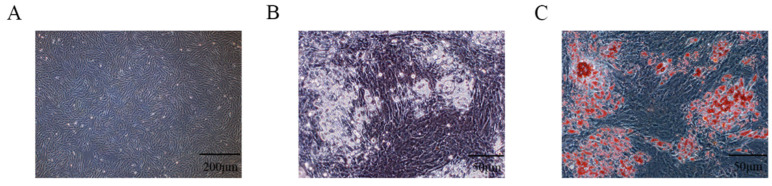
Isolation and identification of PIMPA. (**A**). PIMPA proliferation reached 80% fusion (200 μm). (**B**). Intracellular lipid droplets under microscope after adipogenic induction (scale bar = 50 μm); (**C**). ORO positively stained cells to identify lipid (scale bar = 50 μm).

The longissimus dorsi muscle tissue was aseptically separated, which was then digested by collagenase type I to isolate PIMPA, and then the PIMPA was purified by differential adhesion method. The PIMPA was plated into the cell culture plates at a density of 3 × 10^4^ cells/cm^2^; after growing 4 days, the density of PIMPA reached 85%, showing short fusiform or irregular small triangles, similar to fibroblasts, with no lipid drops in the cytoplasm (Figure 1A). When PIMPA reached 85–90% confusion, “cocktail method” (0.5 mM 3-IBMX, 1 μM DEX, and 1.7 μM insulin) was used to induce adipogenic differentiation. After adipogenic induction of DEX, IBMX, and insulin for 7 days, most of the PIMPA had differentiated into mature adipocytes, and a large number of lipid droplets could be seen under the microscope (Figure 1B). ORO staining method was also used to identify the cellular lipid accumulation, and results showed that a mass of ORO-stained cells were observed, showing that PIMPA underwent differentiation into mature adipocytes following treatment with differentiation-inducing solution (Figure 1C). The above results suggested that PIMPA was successfully isolated and could be used for further analysis.

### 3.2. MiR-130b Overexpression Significantly Promoted the Proliferation of PIMPA

**Figure 2:**
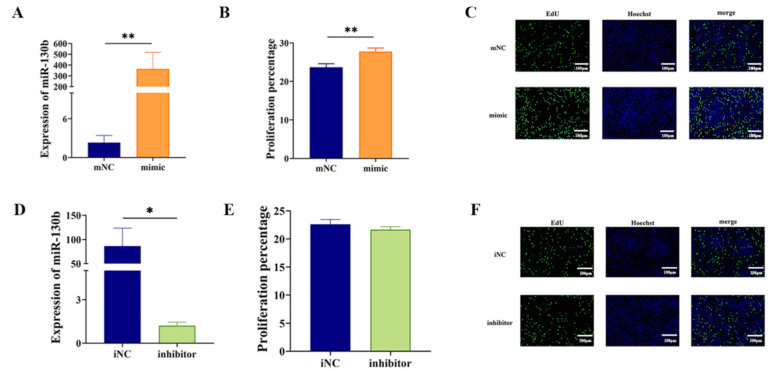
Effect of miR-130b mimic and inhibition on PIMPA proliferation. (**A**). miR-130b expression. (**B**). Cell proliferation rate by Image J (V1.8.0.112) method. (**C**). Proliferative activity by EdU method (scale bar = 100 μm). (**D**). miR-130b expression. (**E**). Cell proliferation rate by Image J method. (**F**). Proliferative activity by EdU method (scale bar = 100 μm). Values are presented as the mean ± SEM. ** Represents *p* < 0.01 mNC vs. miR-130b mimic; * Represents *p* < 0.05 iNC vs. miR-130b inhibitor.

The RT-qPCR analysis indicated that, after transfected with miR-130b mimic, miR-130b expression was significantly elevated in PIMPA (*p* < 0.01) (Figure 2A). The EdU staining result revealed a significant increase in both the number and fluorescence intensity of proliferating green fluorescent cells in the miR-130b mimic group compared to control group. This finding suggests that miR-130b mimic markedly enhanced the proliferation of EdU-labeled preadipocytes. (Figure 2B,C). On the other hand, transfection with the miR-130b inhibitor resulted in reduced miR-130b expression compared to iNC group (*p* < 0.05) (Figure 2D). These results suggest that miR-130b mimic and miR-130b inhibitor were successfully transfected into PIMPA. The proliferation activity assay results indicated that miR-130b mimic significantly enhanced the viability of PIMPA. However, the EdU staining result indicated that miR-130b inhibitor showed no significant difference compared with iNC group (Figure 2E,F).

### 3.3. Overexpression of miR-130b Promoted the Expression of PCNA and cyclinb1 and Inhibited the Expression of P27 in PIMPA

**Figure 3:**
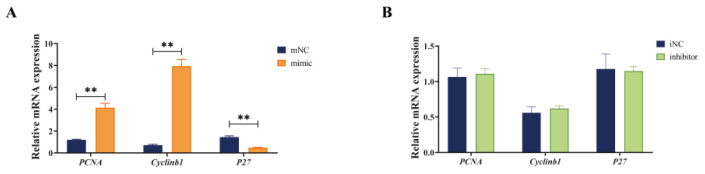
Effect of miR-130b mimic and inhibition on cell cycle-related gene expression of PIMPA. (**A**). Effect of miR-130b mimic on expression of cell cycle-related gene PCNA, cyclinb1, and P27. (**B**). Effect of miR-130b inhibition on expression of PCNA, cyclinb1, and P27. Values are presented as the mean ± SEM. ** Represents *p* < 0.01 mNC vs. miR-130b mimic.

The RT-qPCR results showed that compared with mNC group, miR-130b mimic significantly upregulated the expression of proliferation marker genes proliferating cell nuclear antigen (*PCNA*) and *cyclinb1*, while downregulating the *P27* mRNA expression at day 2 during adipogenic differentiation of PIMPA (*p* < 0.01) (Figure 3A). On the other hand, miR-130b inhibitor showed no significant difference in *PCNA*, *cyclinb1*, and *P27* compared with iNC group (Figure 3B). These findings indicated that miR-130b overexpression markedly increased the PIMPA proliferation through the cell cycle and DNA replication pathways.

### 3.4. Overexpression of miR-130b Significantly Inhibited the Adipogenic Differentiation of PIMPA

**Figure 4:**
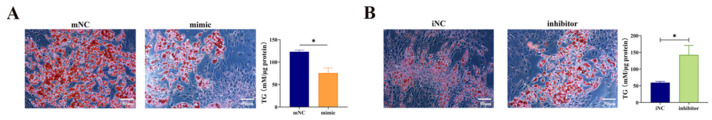
Effect of miR-130b mimic and inhibition on adipogenic differentiation of PIMPA. (**A**). Effect of miR-130b mimic on lipid accumulation and intracellular TG content by ORO staining and TG quantitative detection kit. (**B**). Effect of miR-130b inhibition on lipid accumulation and intracellular TG content by ORO staining and TG quantitative detection kit. Scale bar = 50 μm. Values are presented as the mean ± SEM. * Represents *p* < 0.05 mNC vs. miR-130b mimic.

Both ORO staining and TG quantitative detection kit were used to detect the effect of miR-130b on the adipogenic differentiation of PIMPA. The results depicted in Figure 4 confirm the successful transfection of miR-130b mimic, miR-130b inhibitor, mNC, and iNC into PIMPA by day 7. Additionally, ORO staining revealed that miR-130b mimic markedly reduced lipid droplet accumulation and intracellular TG content compared to the mNC group (Figure 4A). Conversely, transfection with miR-130b inhibitor led to a significant increase in both lipid droplet accumulation and intracellular TG content (Figure 4B). Quantification of the ORO staining area further validated these findings, demonstrating that the miR-130b mimic significantly reduced the stained area compared to mNC group (Figure 4A). In contrast, miR-130b inhibitor increased the area of ORO staining (Figure 4B). In addition, the results of quantitative detection of intracellular TG content were consistent with those of ORO staining results, showing that miR-130b mimic significantly reduced the intracellular TG content by 50% (Figure 4A), while miR-130b inhibitor significantly increased the intracellular TG content by 80% (Figure 4B). These findings indicate that miR-130b acts as a negative regulator of adipogenic differentiation in PIMPA.

### 3.5. Overexpression of miR-130b Significantly Inhibited the Expression of Key Genes for Adipogenesis

**Figure 5:**
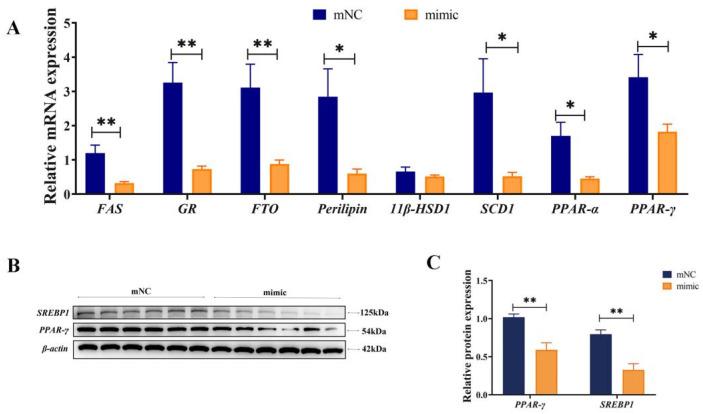
Effect of miR-130b mimic on the expression of key adipogenic genes of PIMPA. (**A**). Relative mRNA expression of FAS, GR, FTO, Perilipin, 11β-HSD1, SCD-1, PPAR-α, and PPAR-γ. (**B**,**C**). Relative protein expression of SREBP-1 and PPAR-γ. Values are presented as the mean ± SEM. * Represents *p* < 0.05; ** Represents *p* < 0.01 mNC vs. miR-130b mimic. The original images of the Western blot are published as Supplementary Materials—Figure S1–S3.

On day 7 after transfection with miR-130b mimic, qPCR and Western blot were employed to analyze the expression levels of essential genes related to lipid metabolism. The results showed that compared with mNC, the mRNA expression of adipogenic differentiation marker genes PPAR-γ, FTO, and GR; lipogenic genes FAS and SCD-1; Perilipin (a critical regulator of fat storage and breakdown); and lipolytic gene PPAR-α were all significantly decreased in miR-130b mimic group (*p* < 0.05) (Figure 5A). In addition, Western blot results also showed that the protein expression of PPAR-γ and SREBP1 were markedly reduced (*p* < 0.01) (Figure 5B,C). The above results showed that miR-130b mimic significantly inhibited the adipogenic differentiation of PIMPA through reducing PPAR-γ and its downstream de novo fatty acid synthesis.

### 3.6. miR-130b Inhibition Significantly Promoted the Expression of Key Genes for Adipogenesis

**Figure 6:**
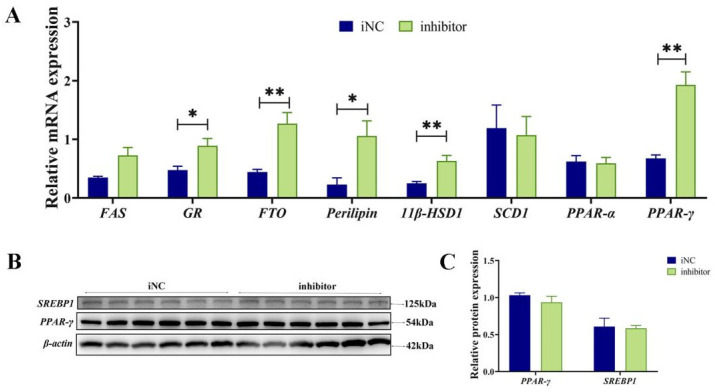
Effect of miR-130b inhibition on the expression of key adipogenic genes of PIMPA. (**A**). Relative mRNA expression of FAS, GR, FTO, Perilipin, 11β-HSD1, SCD-1, PPAR-α, and PPAR-γ. (**B**,**C**). Relative protein expression of PPAR-γ and SREBP-1. Values are presented as the mean ± SEM. * Represents *p* < 0.05; ** Represents *p* < 0.01 iNC vs. miR-130b inhibitor. The original images of the Western blot are published as Supplementary Materials, Figure S1–S3.

On the contrary, on day 7 after transfection with miR-130b inhibitor, qPCR and Western blot results showed that compared to iNC group, the mRNA expression of adipogenic differentiation marker genes PPAR-γ, FTO, GR, FAS, SCD-1, Perilipin, and PPAR-α were all significantly increased in miR-130b inhibitor group (*p* < 0.05) (Figure 6A). However, protein expression of PPAR-γ and SREBP1 showed no significant difference between the two groups (Figure 6B,C). These results demonstrated that miR-130b inhibition significantly up-regulated mRNA levels of lipid metabolism and thus promoted adipogenic differentiation of PIMPA.

## 4. Discussion

Genetic selection of livestock and poultry breeds has mainly focused on increasing growth rate and lean meat percentage in the past few decades, and a large number of candidate genes had received strong selection to improve livestock performance. However, the rapid growth is inevitably accompanied by the reduction of IMF deposition, which eventually leads to the deterioration of livestock and poultry meat quality. Previous studies have proven that in longissimus dorsi muscle of Laiwu pigs with significantly different IMF content, miRNAs were differentially expressed, which were mainly concentrated in lipid metabolism, lipid storage, Wnt, mTOR, and PPAR signaling pathways by GO and KEGG analysis, suggesting that differentially expressed miRNAs played a vital role in regulating pig IMF content [22]. Moreover, a growing number of reports showed that miRNAs play important roles in controlling preadipocyte proliferation and adipogenesis in livestock animals. However, this is the first study to systematically report the effect of miR-130b on proliferation and differentiation of porcine preadipocytes, suggesting that miR-130b can serve as a candidate gene to regulate porcine intramuscular fat deposition and enhance meat quality.

Previous study has shown that miR-130b participates in the inhibition of mouse muscle cell line C2C12 proliferation and promotes myogenic differentiation by targeting Sp1 [23]. Moreover, miR-130b was involved in oxidative metabolism of muscle lipid via targeting PGC-1α [13]. The above results have fully demonstrated the vital role of miR-130b in the development and metabolism of muscle tissue. A previous study in goat has demonstrated that miR-130b-3p/miR-130b-5p duplex reduced adipogenesis by directly inhibiting KLF3 expression, suggesting that KLF3 is a candidate gene of miR-130b [17]. Therefore, the expression of KLF3 after miR-130b mimic or inhibitor treatment should be detected in our future study. However, in the study, we found that miR-130b mimic significantly reduced the mRNA and protein expression of PPAR-γ, one of the most important transcription factors regulating the differentiation of all adipocytes; furthermore, PPAR-γ downstream factors, FAS, GR, FTO, Perilipin and SREBP-1, were all downregulated. Moreover, in our previous studies, we have verified that PPAR-γ is a target of miR-130b [19,20,21]. Moreover, miR-301b~miR-130b-PPARγ axis has been proved to play an important role in MSCs adipogenesis [24]. Therefore, in the study, we could conclude that miR-130b inhibited adipogenic differentiation of PIMPA by blocking PPAR-γ expression, thus dominating IMF deposition in pigs.

Numerous previous studies have demonstrated that miR-130b functions in regulating the proliferation process of various cells [25]. However, we provided the first evidence demonstrating that miR-130b enhanced the proliferation of PIMPA, characterized by increased cell viability and EdU-labeled cell density of PIMPA, as well as increased expression of proliferation marker genes Cyclinb1 and PCNA, and inhibited expression of P27. These results were in accordance with previous study findings that miR-429 mimics could, respectively, promote the proliferation of porcine preadipocytes [26]. It is known that reduced cell proliferation is related to arrested cell cycle-dependent mechanisms, which is strictly controlled by an array of cyclins and cyclin-dependent kinases (CDKs), among which p27 is one of the broad-spectrum CDKs inhibitors, negatively regulating the transition from G1 to S phase of cell cycle. In the study, we found that miR-130b mimic markedly downregulated P27 expression, suggesting that miR-130b promoted proliferation of PIMPA by blocking the G1/S phase transition and thus negatively arrested the cell cycle. Furthermore, Cyclinb1 has been shown to accelerate the proliferation and promote cell cycle of various cell types by moving from G1 to S phase. PCNA has also been shown to play a vital role in cell proliferation, and has been considered as a potential target of cancer therapy [27]. Our current results indicated for the first time that miR-130b promoted proliferation of PIMPA by regulating expression of PCNA, cyclinb1, and P27, which was consistent with previous findings in many types of cells, showing enhanced effect of miR-130b on proliferation. However, which target gene was involved in miR-130b regulating the cell cycle needs to be further studied. Moreover, some other studies have shown diametrically opposite results concerning the effect of miR-130b on proliferation, based on the inhibition role of miR-130b in proliferation. The above results suggested that different role of miR-130b in regulating cell proliferation was closely related to cell types.

Numerous studies have demonstrated that PPAR-γ plays a vital role in lipid accumulation. In order to know whether miR-130b reduced lipid accumulation is related to changes of PPAR-γ signaling pathway, we further conducted the expression of PPAR-γ and its downstream target genes in differentiated PIMPA, and the results showed that miR-130b mimic decreased the accumulation of lipid droplets and expression of PPAR-γ, FTO, GR, FAS, and SCD-1, suggesting that miR-130b might reduce lipid accumulation of PIMPA by repressing the expression of PPAR-γ and its downstream target genes. This was further validated by ORO staining and RT-qPCR experiments following the overexpression or inhibition of miR-130b in PIMPA. These results are consistent with our previous study, which demonstrated that miR-130b overexpression inhibited lipid accumulation and differentiation in primary cultured preadipocytes derived from subcutaneous adipose tissue. miR-130b overexpression inhibited lipid accumulation and differentiation of primary cultured subcutaneous adipose tissue-derived preadipocytes [20]. It is known that PPAR-γ, FTO, GR, FAS, and SCD-1 expression were positively associated with the extent of adipocyte differentiation and have been identified as essential genes for lipogenesis. For example, PPAR-γ is a marker gene of adipogenic differentiation, acting as a molecular switch in the process of adipocyte differentiation [28]. Numerous studies have demonstrated that PPAR-γ overexpression significantly induced lipid transport and TG synthesis in adipocytes, and ultimately resulted in the hypertrophy of adipose tissue [28]. In the study, miR-130b mimic decreased the expression of PPAR-γ as well as its downstream target genes related to adipogenesis and lipogenesis, including FAS and GR. It was demonstrated that PPAR-γ was able to accelerate the activation of adipocyte-specific genes and activate the expression of lipid-metabolizing enzymes, such as aP2, LPL, and FAS [29]. Furthermore, the expression of SREBP-1c, also known as adipocyte differentiation-dependent factor 1 (ADD1), was also inhibited in miR-130b mimic cells. Previous studies have shown that as a transcription factor, SREBP-1c was able to promote PPAR-γ expression and the production of endogenous PPAR-γ ligands [30]. Furthermore, SREBP-1c can also directly increase the expression of lipogenic genes, such as ACC, FAS, and SCD-1 [31]. Therefore, we can sum up that decreased PPAR-γ and SREBP-1c resulted in repressed expression of FAS and GR. Consequently, our current data demonstrated that miR-130b significantly suppressed adipocyte differentiation and lipid accumulation in PIMPA through inhibiting adipogenesis-related genes in mature adipocytes.

It has been well reported that lipid droplet-associated proteins, locating on the surface of lipid droplets, are correlated with lipid accumulation and participate in regulating lipid metabolism, leading to trigger activation or inhibition signaling of lipolysis [32]. Lipolysis and perilipin-1 are correlated with lipid accumulation. Perilipin-1, the predominant lipid droplet-associated protein, serves as a key regulatory gatekeeper in controlling intracellular lipolysis in adipocytes under basal conditions [33]. Perilipin-1 interacts with lipid droplets, forming a protective barrier that restricts lipase access, thereby reducing lipase-mediated TG hydrolysis and promoting TG storage [34]. In the study, both PPAR-γ and Perilipin-1 were significantly inhibited in miR-130b mimic cells compared with mNC group. Numerous studies have demonstrated that a PPAR-responsive element has been identified within the Perilipin-1 gene. Therefore, PPAR-γ regulates Perilipin-1 protein expression by binding to the Perilipin-1 gene promoter, thereby increasing Perilipin-1 levels. Further studies showed that two different PPAR-γ activators (darglitazone and rosiglitazone) could induce the expression of Perilipin-1 in adipocytes [35]. Therefore, our results suggested that miR-130b mimic resulted in inhibition of PPAR-γ, which thus led to decreased Perilipin-1 expression and activated lipolysis.

Numerous studies have demonstrated that FTO is a key regulator of adipogenesis. APrevious research showed that FTO promoted adipogenesis through inhibiting Wnt/β-catenin signaling pathway in PIMPA [36], which is consistent with our result that miR-130b significantly inhibited FTO expression and thus inhibited adipogenic differentiation of PIMPA. In addition, FTO overexpression in differentiating PIMPA also significantly increased the mRNA expression of PPAR-γ and FAS [37]. Our findings provided the first functional evidence demonstrating the role of miR-130b in PIMPA differentiation by regulating FTO expression. Moreover, a genome-wide study identified the FTO gene on porcine chromosome 6, a region potentially harboring genes associated with meat quality traits, including IMF content [38]. Moreover, the porcine FTO (pFTO) gene has been extensively reported to be linked with fat-related traits, such as IMF deposition, IMF content, and the total lipid percentage in muscle [39]. These above data suggest a novel role of miR-130b in regulating both PIMPA differentiation and proliferation, with PPAR-γ identified as a potential target of miR-130b in these processes. Furthermore, due to the physiological and anatomical similarities between pigs and humans, as well as the resemblance in adipocyte lipogenic patterns, miR-130b may also play a crucial role in human obesity treatment. Therefore, further investigations into the function of miR-130b in human preadipocytes are warranted.

## 5. Conclusions

In summary, miR-130b overexpression accelerated the proliferation of PIMPA by obviously promoting cell cycle progression, while inhibiting adipogenic differentiation by dramatically reducing expression of PPAR-γ and its downstream target genes. This study contributes to understanding the roles of miR-130b in the proliferation and differentiation of PIMPA, and it also provides a theoretical platform for improving fat content by manipulating the expression of miR-130b in pigs.

## Figures and Tables

**Table 1:** Primer sequence of qPCR reaction.

Gene Name	GenBank		Primer Sequence (5′-3′)	Annealing Temperature (°C)	PCR Product (bp)
Porcine					
*P27*	NM_214316.1	F	GTCCCTTTCAGTGAGAACCGATAC	61	134
		R	TTGCTGCCACATAACGGAATCAT		
*PCNA*	NM_001291925.1	F	GTGATTCCACCACCATGTTC	57.9	145
		R	TGAGACGACTCCATGCTCTG		
*Cyclinb1*	NM_001170768.1	F	AACTGCTCTTGGAGACATCGGT	61.8	196
		R	TGGTTCAGGCTCCAGTTCAGG		
*FAS*	NM_001099930.1	F	GTCCTGCTGAAGCCTAACTC	57.2	206
		R	TCCTTGGAACCGTCTGTG		
*GR*	NM_001008481.1	F	CCAAACTCTGCCTTGTGTGTTC	61	108
		R	TGTGCTGTCCTTCCACTGCT		
*FTO*	NM_001112692.1	F	GGAGAAAGCCAATATCGACACC	60.1	109
		R	TCTGCTCTTCCTGTCCACCTC		
*Perilipin*	NM_001038638.1	F	GCCTGACTTTGCTGGATGG	60.5	119
		R	CTTGGTGCTGGTGTAGGTCTTCT		
*11β-HSD1*	NM_214248.3	F	CCATGCTGAAGCAGAGCAAC	59.6	115
		R	AAGAACCCGTCCAGAGCAAA		
*SCD1*	NM_213781.1	F	CCCAGCCGTCAAAGAGAA	56.5	200
		R	CGATGGCGTAACGAAGAAA		
*PPAR-α*	NM_001044526.1	F	GAGCCTGAGGAAACCCTTCT	58.2	128
		R	GGTCTCCGCACCAAATGA		
*PPAR-γ*	NM_214379.1	F	GCCCTTCACCACTGTTGATT	58.5	210
		R	GAGTTGGAAGGCTCTTCGTG		
*C* *aspase-3*	NM_214131.1	F	TGCTGCAAATCTCAGGGAGACCT	64.6	288
		R	GTGCCTCGGCAGGCCTGAAT		
*PPIA*	NM_214353.1	F	AGGATTTATGTGCCAGGGTG	50.9	126
		R	ATGGACAAGATGCCAGGAC		

**Table 2:** Antibody information in the study.

Antibody Name	Species Source	Number	Company
PPAR-γ	Rabbit polyclonal antibody	BS6442	Bioworld (Shanghai, China)
β-actin	Rabbit polyclonal antibody	AP0060	Bioworld (Shanghai, China)
SREBP1	Rabbit polyclonal antibody	14088-1-AP	Proteintech (Wuhan, China)
Caspase-3	Rabbit polyclonal antibody	19677-1-AP	Proteintech (Wuhan, China)
Bcl-2	Rabbit polyclonal antibody	26593-1-AP	Proteintech (Wuhan, China)

**Table 3:** miR-130b related sequences.

Name	Sequences (5′-3′)
miR-130b mimic	CAGTGCAATGATGAAAGGGCAT
	GCCCTTTCATCATTGCACTGTT
mimic NC	TTCTCCGAACGTGTCACGTTT
	ACGTGACACGTTCGGAGAATT
miR-130b inhibitor	ATGCCCTTTCATCATTGCACTG
inhibitor NC	CAGTACTTTTGTGTAGTACAA
Universal primer	TAGAGTGAGTGTAGCGAGCA
External reference	GTGACCCACGATGTGTATTCGC
Poly (T) adapter	TAGAGTGAGTGTAGCGAGCACAGAATTAATACGACTCACTATAGGTTTTTTTTTTTTTTTTVN

## Data Availability

The datasets analyzed during the current study are available in this paper and Supplementary Materials.

## Supplementary Materials

The following supporting information can be downloaded at: https://www.mdpi.com/article/10.3390/vetsci12040375/s1, Figures S1–S3: The original image of the Western blot.

## Abbreviations

ACC	acetyl-CoA carboxylase
DEPC	diethylpyrocarbonate
FAS	fatty acid synthesis
FTO	fat mass and obesity-associated protein
GR	glucocorticoid receptor
IMF	intramuscular fat
miRNAs	microRNAs
ORO	oil red o
PCNA	proliferating cell nuclear antigen
PIMPA	porcine primary intramuscular preadipocytes
PPAR-α	peroxisome proliferator activated receptor-α
PPAR-γ	peroxisome proliferator-activated receptor-gamma
PPIA	peptidylprolyl isomerase A
RT-qPCR	reverse transcription-quantitative polymerase chain reaction
SCD-1	stearyl-CoA-desaturase-1
SREBP-1	sterol regulatory element binding protein-1
TNF	tumor necrosis factor
11β-HSD1	11 beta-hydroxysteroid dehydrogenase type 1
