# Supplementary material for: Ssc-miR-130b Enhances Cell Proliferation and Represses Adipogenesis of Primary Cultured Intramuscular Preadipocytes in Pigs
Source: Vet Sci. 2025 Apr 17;12(4):375. doi: 10.3390/vetsci12040375 (PMC12030777; doi:10.3390/vetsci12040375)
Supplement: Supplementary file 1 [file vetsci-12-00375-s001.zip › vetsci-3516845-supplementary.pdf]

1. **Orginal gels of SREBP-1 and PPAR-γ proteins between mimic and mNC groups (N=6/group)**

A. Molecular weight standard of protein marker provided in the instruction manual

B. Orginal gels of SREBP-1 (125kDa) and PPAR-γ (54kDa) proteins with protein markers

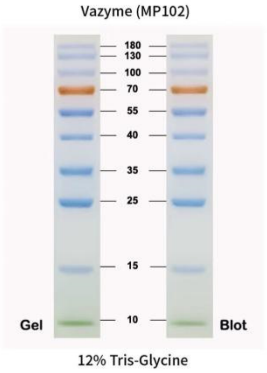

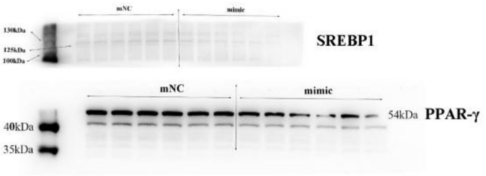

2. **Orginal gels of SREBP-1 and PPAR-γ between inhibitor and iNC groups (N=6/group)**

A. Molecular weight standard of protein marker provided in the instruction manual

B. Orginal gels of SREBP-1 (125kDa) and PPAR-γ (54kDa) proteins with protein markers

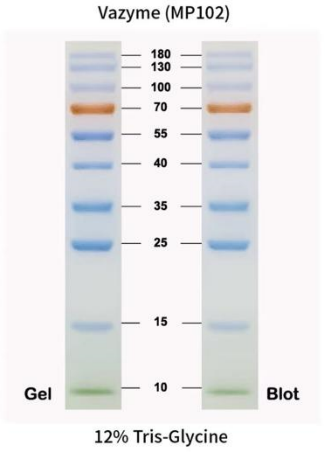

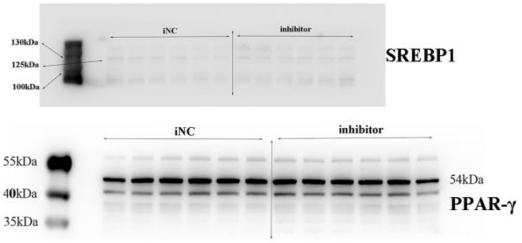

3. Estimated molecular weight of SREBP-1 antibody

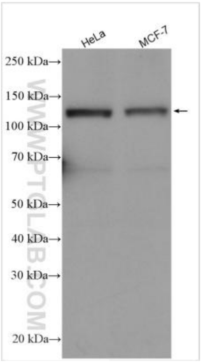

Estimated molecular weight provided in the instruction of SREBP-1 antibody
